# Ghanaian women’s knowledge on whether malaria treatment is covered by the national health insurance: A multilevel regression analysis of national data

**DOI:** 10.1186/s12889-021-12290-7

**Published:** 2021-12-11

**Authors:** Edward Kwabena Ameyaw, Linus Baatiema, Bright Opoku Ahinkorah, Abdul-Aziz Seidu, Jerry Paul Ninnoni, John Kuumuori Ganle

**Affiliations:** 1 School of Public Health, Faculty of Health, University of Technology Sydney, Sydney, Australia; 2 L & E Research Consult Limited, Wa, Ghana; 3 Department of Estate Management, Takoradi Technical University, Takoradi, Ghana; 4 College of Public Health, Medical and Veterinary Sciences, James Cook University, Townsville, Queensland Australia; 5 School of Nursing and Midwifery, University of Cape Coast, Townsville, Ghana; 6 Department of Population, Family and Reproductive Health; School of Public Health, University of Ghana, Accra, Ghana

**Keywords:** Malaria, National health insurance scheme, Women, Healthcare, Public health, Ghana

## Abstract

**Background:**

To obviate malaria and other healthcare costs and enhance healthcare utilization, the government of Ghana introduced the National Health Insurance Scheme (NHIS) in 2005. Nonetheless, there is dearth of empirical evidence on Ghanaian women’s knowledge about whether malaria treatment is covered by the NHIS or not. The current study, therefore, investigated factors associated with knowledge of malaria treatment with the NHIS among women aged 15-49 in Ghana.

**Methods:**

The study is a secondary analysis of data from women respondents in the 2014 Ghana Demographic and Health Survey. A total of 2,560 women participated in this study. Descriptive computation of the weighted proportion of women who knew that malaria is covered by NHIS was conducted at 95% confidence interval (CI). A multilevel logistic regression analyses was carried out with Stata’s MLwinN package version 3.05. We declared significance at 5% alpha. Findings from the models were reported as adjusted odds ratios (aOR) and credible intervals (CrIs).

**Results:**

In all, 81.0% of Ghanaian women included in the study knew that NHIS covers malaria treatment. Women aged 45-49 had higher odds of knowing that NHIS covers malaria relative to those aged 15-19 age category [aOR=1.5;95%crl=1.2-2.1]. Women with higher education (post-secondary) had higher odds of knowing that NHIS covers malaria treatment compared with women who had no formal education [aOR=1.6;95%Crl=1.2-2.0]. Richest women were more likely to know that NHIS covers malaria treatment compared to the poorest women [aOR=1.3;95%Crl=1.2-1.7]. Women who had subscribed to the NHIS were more likely to report that NHIS covers malaria treatment [aOR=1.5;95%Crl=1.2-1.8]. The study revealed that the variance in the tendency for a woman to be aware that NHIS covers malaria treatment is attributable to 10.8% community level factors.

**Conclusion:**

This study has shown that individual, community and regional level factors affect women’s knowledge on whether NHIS covers malaria treatment or not. As knowledge that malaria treatment is covered by NHIS may increase use of malaria prevention and treatment services in health facilities, we recommend that the Ghana Health Service intensifies community level education and awareness creation efforts, targeted at women among whom awareness levels are currently low.

## Background

Malaria is a major cause of morbidity and mortality in sub-Saharan Africa (SSA) [1]. In 2015, nearly 88% of global malaria cases and 90% of malaria induced mortalities occurred in SSA [2]. Malaria poses enormous financial burden to individuals, households and national economies in low and middle-income countries [3, 4]. The worse affected are pregnant women and children under five years [5]. The 2019 Global Malaria Report indicates that Ghana’s malaria cases increased by 8% between 2017 and 2018 [6]. Malaria is a leading cause of morbidity in Ghana, accounting for 38% of all outpatient cases, 27.3% of all health facility admissions, and 7.2% of all deaths on admission in 2014 [7]. Ghana was one of the two countries with the highest increase in malaria prevalence in 2018 in Africa [6].

At the household level, US$14.61 expenditure per patient per fever/malaria episode has been recounted from the middle belt of Ghana [8]. Similarly, Dalaba, Akweongo [9] reported an average cost of US$13.9 per malaria treatment for households in Northern Ghana. Regrettably, the poor have the greatest risk of malaria, partly because of poor sanitation, low socio-economic status and poor housing [10]. Due to cost of treatment, poor women may also delay healthcare for malaria [11].

To address malaria and other healthcare costs and enhance healthcare utilization, especially for poor women in the reproductive age, Ghana introduced the National Health Insurance Scheme (NHIS) in 2005 [12]. All subscribers to the scheme are entitled to an array of services including malaria care. The scheme is functional in both accredited private and public health institutions nationwide. The expectation is that women who are aware that malaria treatment is covered by the NHIS are less likely to self-medicate when they develop malaria symptoms and are more likely to report to the health facility for treatment [10]. Further, knowing that malaria treatment is covered by NHIS enhances the uptake of intermittent preventive treatment in pregnancy (IpTP) [13]. While it is expected that women who are subscribed to NHIS will seek formal healthcare for malaria as opposed to informal or self-medication, the increasing malaria cases in Ghana raises questions about access to formal healthcare as well as awareness about the fact that malaria is covered by NHIS [14].

Previous research on Ghana’s NHIS have focused on its sustainability [15] and estimation of economic burden of malaria treatment under the health insurance scheme [9, 16]. The few on awareness and knowledge were limited in geographical scope and have focused on awareness of expiration status [17, 18]. None of the pre-existing studies however examined women’s knowledge on whether NHIS covers malaria treatment at the national level. Additionally, none explored how community and regional level factors affect women’s knowledge on whether NHIS covers malaria treatment. The present study addressed this knowledge gap by examining individual, community and regional level factors associated with whether women are aware that malaria is covered by the NHIS.

## Methods

### Design and data source

The study is a secondary analysis of data from women respondents in the 2014 Ghana Demographic and Health Survey. The women’s recode file from the 2014 Ghana Demographic and Health Survey (GDHS) was utilized. The 2014 GDHS is the sixth wave since 1988. It sought to produce reliable and current information on malaria, maternal and child health, nutrition and several other essential population health information required for informed policy decisions and programme evaluation.

### Sampling procedure

Sampling was carried out with an updated frame from the 2010 Population and Housing Census (PHC). This frame does not include nomadic and institutional populations. To ensure estimation of core indicators at the national level, account for urban/rural settings as well as the ten erstwhile administrative regions, two-stage sampling design was employed. The first stage comprised selection of sample points or clusters made up of enumeration areas (EAs) demarcated during the 2010 PHC. A total of 427 clusters were selected from urban (216) and rural (211) locations. In the second stage, households listing/enumeration was done between January and March 2014. Eligible households were then randomly selected from the list. Approximately, 30 households were selected from each of the clusters culminating in 12,831 households. Interviews were successfully conducted in 11,835 of the selected households (i.e. 92.2% response rate). Within the interview households, 9,656 eligible women aged 15-49 were identified, out of which 9,396 were successfully interviewed (i.e. 97.3% response rate). In the present study however, 2,560 women with complete data are included in the analysis.

### Dependent variable

Women’s awareness that malaria treatment is covered by the national health insurance scheme (NHIS) was the outcome variable. It was derived from the question; “are you aware that malaria treatment is covered under national health insurance?” The responses to this question were either ‘Yes’ =1 or ‘No’=0. All women who responded ‘Yes’ knew that malaria treatment is covered by NHIS whilst ‘no’ response implied otherwise.

### Independent variables

Based on conceptual relevance and conclusions drawn by earlier studies [2, 4, 11, 19], eleven independent variables were considered in the current study. Based on the hierarchical nature of the data, the variables were layered into three levels: individual, community and regional levels. Individual level variables included age in years (15-19, 20-24, 25-29, 30-34, 35-39, 40-44 and 45-49), level of education (no education, primary, secondary and higher), wealth quintile (poorest, poorer, middle, richer and richest) and parity (1, 2, 3 and 4). Other individual level variables were current pregnancy status (no or unsure and yes), NHIS subscription (no and yes), and whether seen or heard malaria message through media (no and yes). Media comprised radio, television and newspaper. Community factors comprised three variables: ethnicity (Akan, Ga/dangme/Ewe, Guan, Mole-dagbani, Grusi, Gurma, Mande and other), sex of family head (male or female), and place of residence (urban and rural). Ecological zone (coastal, middle and savanna), as defined by the Ghana Statistical Service (GSS), was considered as a regional level variable [20].

### Analytical approach

Descriptive computation of weighted proportion of women who knew that malaria treatment is covered by NHIS was conducted at 95% confidence interval (see Table 1). We subsequently specified a three-level multilevel logistic regression with five models, thereby generating fixed-effects and random-effects (see Table 2). The empty model, being the first model (Model 1), was devoid of independent variables and decomposed the magnitude of variance at both the community and regional levels. The second model (Model 2) reflected individual level factors only, third model (Model 3) accounted for community-level factors only whilst the fourth model (Model 4) constituted the regional-level factors only. The ultimate model (Model 5), being the complete model, constituted variables from all four models. The fixed-effects (measures of association) were reported in adjusted Odds Ratios (aOR) at 95% credible interval (CrI). However, the random-effects (measures of variation) were reported as variance partition coefficients (VPC) and median odds ratios (MOR), otherwise known as intra-cluster correlation (ICC). Detailed documentation of VPC and MOR have been carried out elsewhere [21]. Stata version 13 was used for all the analyses.

**Table 1:** Proportion of women who are aware that NHIS covers malaria (2,560)

Variable	Weighted N n(%)	NHIS covers Malaria treatment	
		Yes %(CI)	No %(CI)
	2,560(100%)	n=2,076; 81.0%	n=484; 19.0%
***Age(Years)***			
15-19	129(5.1)	79.0(71.3-85.6)	21.0(14.4-28.6)
20-24	452(17.7)	80.1(76.2-83.5)	19.9(16.4-23.8)
25-29	601(23.4)	80.0(76.0-83.0)	20.0(17.2-23.6)
30-34	628(24.6)	81.6(78.3-84.5)	18.4(15.4-21.7)
35-39	461(18.0)	82.3(78.5-85.5)	17.7(14.4-21.5)
40-44	229(8.8)	83.0(78.0-87.2)	17.0(12.7-22.2)
45-49	60(2.4)	82.7(72.3-89.7)	17.3(10.3-27.7)
***Education***			
No education	649(25.4)	83.0(80.3-85.4)	17.0(14.5-19.6)
Primary	483(18.9)	81.6(77.9-84.8)	18.4(15.2-22.0)
Secondary	1,256(49.1)	79.0(76.5-81.3)	21.0(18.6-23.5)
Higher	172(6.7)	83.5(76.4-88.8)	16.5(11.1-23.5)
***Wealth quintile***			
Poorest	508(19.8)	84.1(81.5-86.4)	15.9(13.5-18.4)
Poorer	513(20.1)	82.5(78.9-85.5)	17.5(14.4-21.1)
Middle	547(21.4)	82.1(78.3-85.4)	17.9(14.6-21.7)
Richer	517(20.2)	77.4(73.1-81.2)	22.6(18.8-26.8)
Richest	475(18.5)	74.5(69.6-78.9)	25.5(21.0-30.3)
***Parity***			
1	1,532(59.8)	81.1(79.1-83.0)	18.9(17.0-20.9)
2	893(34.9)	80.8(78.0-83.2)	19.2(16.7-21.9)
3	128(5.0)	84.1(76.8-89.4)	15.9(10.6-23.2)
4	7(0.3)	63.6(32.5-86.4)	36.4(13.6-67.5)
***Currently pregnant***			
No or unsure	2,317(90.5)	81.0(79.4-82.6)	19.0(17.4-20.6)
Yes	243(9.5)	81.8(76.2-86.3)	18.2(13.7-23.8)
***NHIS subscription***			
No	976(38.2)	76.4(73.6-78.9)	23.6(21.1-26.4)
Yes	1,583(61.8)	84.0(82.1-85.7)	16.0(14.3-17.9)
***Seen/heard malaria message via media***			
No	1,635(63.9)	82.2(80.4-84.0)	17.8(16.0-19.6)
Yes	925(36.1)	78.7(75.8-81.3)	21.3(18.6-24.2)
**Community factors**			
***Ethnicity***			
Akan	1,229(48.0)	76.9(74.1-79.5)	23.1(20.5-25.9)
Ga/dangme	139(5.4)	76.0(66.8-82.8)	24.0(17.2-33.2)
Ewe	283(11.1)	85.8(81.3-89.3)	14.2(10.6-18.7)
Guan	68(2.6)	86.4(77.4-92.1)	13.6(7.8-22.5)
Mole-dagbani	528(20.6)	85.2(82.3-87.6)	14.8(12.4-17.6)
Grusi	59(2.3)	85.5(77.8-91.0)	14.4(9.0-22.2)
Gurma	160(6.3)	82.0(76.2-86.7)	18.0(13.3-23.8)
Mande	21(0.8)	84.2(68.8-92.8)	15.8(7.2-31.2)
Other	73(2.8)	67.5(56.3-77.1)	32.5(22.9-43.7)
***Sex of Family head***			
Male	1,900(74.2)	82.0(80.2-83.6)	18.0(16.3-19.7)
Female	660(25.8)	78.1(74.6-81.2)	21.9(18.7-25.4)
***Residence***			
Urban	1,198(46.8)	77.1(74.4-79.6)	22.9(20.4-25.6)
Rural	1,362(53.2)	83.7(81.8-85.5)	16.3(14.5-18.2)
***Regional level factor***			
**Ecological zone**			
Coastal	882(34.4)	70.7(67.1-74.0)	29.3(25.9-32.9)
Middle	1,162(45.4)	83.0(80.5-85.1)	17.0(14.9-19.5)
Savanna	516(20.2)	86.8(84.4-88.9)	13.2(11.1-15.6)

Source: 2014 GDHS|| CI: Confidence Interval, NHIS: National Health Insurance Scheme

**Table 2:** Individual, community and regional level predictors of women’s awareness that malaria is covered by NHIS

	Model 1	Model 2	Model 3	Model 4	Model 5
		aOR [95% CrI]	aOR [95% CrI]	aOR [95% CrI]	aOR [95% CrI]
**Fixed-effects**					
***Individual level factors***					
**Age (Years)**					
15-19		1[1,1]			1[1,1]
20-24		1.1**[1.1-1.9]			1.1**[1.1-1.2]
25-29		1.1*[1.10-1.8]			1.1[0.7-1.8]
30-34		1.3**[1.1-2.1]			1.3*[1.1-1.7]
35-39		1.4[0.9-2.3]			1.3**[1.1-2.3]
40-44		1.5[0.9-2.6]			1.4***[1.4-1.5]
45-49		1.5**[1.2-3.2]			1.5***[1.2-2.1]
**Education**					
No education		1[1,1]			1[1,1]
Primary		1.1**[1.1-1.6]			1.1*[1.1-1.2]
Secondary		1.1*[1.0-1.5]			1.1[0.8-1.4]
Higher		1.6**[1.2-1.8]			1.6**[1.2-2.0]
**Wealth quintile**					
Poorest		1[1,1]			1[1,1]
Poorer		1.2***[1.1-1.7]			1.3*[1.2-1.4]
Middle		1.3*[1.2-2.1]			1.5**[1.3-1.9]
Richer		1.1[0.7-1.7]			1.2*[1.1-1.9]
Richest		0.8*[0.6-0.9]			1.3**[1.2-1.7]
**Parity**					
1		1[1,1]			1[1,1]
2		0.7*[0.5-0.9]			0.9[0.7-1.2]
3		1.1**[1.0-1.2]			1.11[0.7-1.8]
4		0.3**[0.1-0.4]			0.3[0.1-1.0]
**Currently pregnant**					
No or unsure		1[1,1]			1[1,1]
Yes		0.9[0.7-1.4]			0.9[0.6-1.5]
**NHIS subscription**					
No		1[1,1]			1[1,1]
Yes		1.5***[1.2-1.8]			1.5***[1.2-1.8]
**Seen/heard malaria message via media**					
No		1[1,1]			1[1,1]
Yes		0.9[0.8-1.2]			0.9[0.7-1.3]
***Community level***					
**Ethnicity**					
Akan			1[1,1]		1[1,1]
Ga/dangme			1.1[0.6-2.0]		1.2[0.7-2.1]
Ewe			1.4[0.8-2.2]		1.4[0.9-2.2]
Guan			1.2[0.6-2.4]		1.2[0.5-2.5]
Mole-dagbani			0.8[0.6-1.30]		0.8[0.6-1.3]
Grusi			0.9[0.5-1.7]		0.9[0.5-1.7]
Gurma			0.8[0.5-1.4]		0.9[0.5-1.6]
Mande			0.85[0.5-2.2]		0.8[0.3-2.3]
Other			0.5*[0.3-0.8]		0.5**[0.3-0.8]
**Sex of Family head**					
Male			1[1,1]		1[1,1]
Female			0.8[0.70-1.1]		0.9[0.7-1.2]
**Residence**					
Urban			1[1,1]		1[1,1]
Rural			1.3[0.9-1.7]		1.3***[1.2-2.1]
***Region level***					
**Ecological zone**					
Coastal				1[1,1]	1[1,1]
Middle				2.0**[1.2-3.4]	1.8**[1.1-3.0]
Savanna				2.8***[1.6-4.9]	3.1***[1.7-5.8]
**Random-effects**					
**Region level**					
Variance [95% CrI]	0.3[0.1-0.5]	0.5[0.2-0.7]	0.3[0.1-0.6]	0.4[0.2-0.7]	0.7[0.3-0.9]
VPC % [95% Crl]	7.0[0.3-12.8]	8.3 [2.9-10.3]	7.5[0.3-13.6]	2.3[0.6-4.8]	2.0[0.9-4.2]
MOR [95% CrI]	1.6[1.10-2.0]	1.7[1.3-1.8]	1.7[1.1-2.0]	1.3[1.1-1.5]	1.3[1.2-1.5]
Explained variation (%)	1[1,1]	19.2[17.8-21.2]	32.3[16.9-41.2]	69.2[65.4-72.9]	73.1[69.2-78.7]
**Community level**					
Variance [95% CrI]	0.2[0.1-0.3]	0.2[0.1-0.3]	0.1[0.1-0.3]	0.2[0.1-0.3]	0.19[0.1-0.3]
VPC % [95% Crl]	10.8[0.6-19.2]	12.3[3.5-17.3]	11.6[8.9-20.1]	7.1[1.5-13.0]	7.3[2.4-13.2]
MOR [95% CrI]	1.4[1.1-1.6]	1.4[1.1-1.7]	1.5[1.1-1.6]	1.5[1.18-1.7]	1.5[1.2-1.7]
Explained variation (%)	1[1,1]	7.1[3.5-9.2]	15.2[10.9-26.9]	21.4[19.2-26.4]	35.7[30.8-44.2]
**Sample Size**					
Region level	10	10	10	10	10
Community level	200	200	200	200	200
Individual level	2,560	2,560	2,560	2,560	2,560

Source: 2014 GDHS

aOR=adjusted Odds Ratio; OR= Odds Ratio; CrI=Credible Interval; VPC= Variance Partition Coefficient; MOR=Median Odds Ratio; 1 = reference; *p < 0.05, **p < 0.01, ***p < 0.001

### Model fit and specifications

Prior to modelling, we investigated the presence of multicollinearity with the Variance Inflation Factor (VIF) and the outcome indicated that none of the  independent variables were highly correlated (mean=1.82; minimum=1.24; maximum=3.15). We used the Bayesian Deviance Information Criterion (DIC) to assess the goodness of fit of the models. The Markov Chain Monte Carlo (MCMC) guesstimate was factored in all the models [22]. Statistical significance was set at 95% confidence interval (CI) and all the modelling operations were conducted with MLwinN package version 3.05.

### Ethical considerations

The survey protocols for the 2014 GDHS were reviewed and approved by the Ethical Review Committee of the Ghana Health Service and the Institutional Review Board (IRB) of Inner-City Fund (ICF) International. Further, informed consent was sought from each participant prior to interview. The ethics processes can be assessed through http://goo.gl/ny8T6X. Authors of this manuscript applied and gained access to the dataset from the MeasureDHS website. The dataset is available to the general public via https://dhsprogram.com/data/dataset/Ghana_Standard-DHS_2014.cfm?flag=0.

## Results

### Descriptive analysis

The proportion of women who knew that NHIS covered malaria treatment is presented in Table 1. The same table reports socio-demographic characteristics of the 2,560 included in the analysis. More women aged 40-44 were aware that NHIS covered malaria treatment (83.0%). Knowing that malaria treatment is covered by NHIS was highest among women who had attained higher education (83.5%) as well as among poorest women (84.1%). Knowledge that malaria treatment is covered by NHIS was also highest among rural women (83.7%) and women at parity three (84.1%). Also, 81.0% of women who were not pregnant or unsure of their pregnancy status knew that NHIS covers malaria treatment, and this did not vary from the proportion of pregnant women who knew that NHIS covers malaria treatment (81.8%). It is worth mentioning that 84.0% of women who had subscribed to the NHIS were aware that it covers malaria treatment. At least eight out of ten of the women who had not seen or heard malaria message through the media knew that NHIS covers malaria treatment (82.2%). Knowledge that NHIS covers malaria treatment in Ghana was common among women of the Guan ethnic group (86.4%). Most women from male-headed households knew that NHIS covers malaria treatment (82.0%). Most women from the Savanna zone indicated that NHIS covers malaria treatment (86.8%).

### Fixed effects (measures of associations)

In the final model (Model 5), women aged 45-49 had higher odds of knowing that NHIS covers malaria treatment relative to those aged 15-19 [aOR=1.5;95%Crl=1.2-2.1]. Compared with women who had no formal education, highly educated women had high propensity of knowing that NHIS covers malaria treatment [aOR=1.6;95%Crl=1.2-2.0]. Compared to the poorest women, the richest women were more likely to know that NHIS covers malaria treatment [aOR=1.3;95%Crl=1.2-1.7]. Relative to urban residents, women in rural locations of Ghana had higher odds of indicating that NHIS covers malaria treatment [aOR=1.3;95%Crl=1.2-2.1]. Compared to those not subscribed to NHIS, women who were subscribed to the NHIS had higher odds of reporting that NHIS covers malaria treatment [aOR=1.5;95%Crl=1.2-1.8]. Compared with women in the Coastal zone, women in the Savanna zone had higher odds of indicating that NHIS covers malaria treatment [aOR=3.1;95%Crl=1.7-5.8].

### Random-effects (measures of variations)

As shown in Table 2, the knowledge that NHIS covers malaria varied by region. VPCs of 10.8% and 7.0% for community and regional level factors respectively suggest that the variation in odds of knowing that NHIS covers malaria treatment is largely attributable to community level factors compared to region level factors. However, results for the MOR indicate that both community and regional level factors explain women’s awareness that NHIS covers malaria treatment in Ghana. The results further show that relocation to a community where higher likelihood of knowing that NHIS covers malaria treatment exist is likely to increase, median awareness that NHIS covers malaria treatment by 1.5%. However in the case of relocation from one region to a different region with high probability, a 1.3% increase will occur.

## Discussion

This study investigated individual, community and regional level factors associated with awareness about whether Ghana’s NHIS covers treatment for malaria among reproductive aged women. Results showed that beyond individual level factors, community and region level factors also affect women’s awareness that malaria treatment is covered by NHIS. This finding highlights that social determinants of health (i.e. conditions in which people are born, grow, work, live, and age, and the wider set of forces and systems shaping the conditions of daily life) [23] are critical to the knowledge of Ghanaian women concerning the benefits associated with NHIS. Different conditions within communities and regions may present varied opportunities to women. These will eventually affect who receives the right information about whether they can access malaria care free of charge under the NHIS. It will therefore be prudent for the National Health Insurance Authority (NHIA) to investigate practical approaches of targeting women in the reproductive age. This will help them to understand that once they are subscribed to NHIS, malaria healthcare can be accessed free of cost.

Women in rural locations had higher odds of indicating that NHIS covers malaria treatment. This is inconsistent with earlier report where higher odds of being unaware that NHIS covers malaria treatment were reported among rural residents [24]. Our finding may be due to the communalism in rural settings, thus the interconnectedness and interpersonal care being held in high esteem in rural settings compared to urban settings [25, 26]. By inference, a woman in rural setting who gets to know that malaria treatment is covered by NHIS is likely to inform other women. In addition to the fact that malaria is more prevalent in rural Ghana [27, 28], rural residents have 1.7 children more than urban residents. These two factors may increase rural women’s contact with healthcare system. This may increase their likelihood to be informed by healthcare providers that NHIS covers malaria than their urban counterparts [29].

Women aged 45-49 had higher odds of knowing that NHIS covers malaria treatment relative to those in the 15-19 age category. All things being equal, as women advance in age, they are more likely to be experienced and knowledgeable in matters pertaining to healthcare [30]. Through childbearing, 45-49 aged women might have interacted severally with healthcare providers through antenatal visits. Frequent interaction with the health system might present an opportunity for women to be informed that NHIS covers malaria treatment. Moreover, childhood malaria is common in Ghana and as women keep seeking healthcare for their children, their chances of being knowledgeable about malaria and NHIS is more probable [31, 32]. This indicates the significant role of maternal experience in women’s prospects of knowing that NHIS covers malaria. Maternal experience accumulates through antenatal care (ANC) visits, health facility-based delivery, postnatal care (PNC) and child healthcare seeking.

Compared with women who had no formal education, highly educated women had higher odds of knowing that NHIS covers malaria treatment. Through education, women gain competencies in reading and have increased social networks. Educated women are also more likely to investigate, and better understand the content of NHIS before subscribing [24]. Consequently, enhancing educational attainment of women may be beneficial to overcome malaria in Ghana.

The richest women were more likely to know that NHIS covers malaria treatment. Wealth usually empowers women, thereby enhancing their chances of attaining knowledge about essential issues concerning their lives [33–35]. This may manifest in improved health knowledge and healthcare seeking behaviour [35, 36]. This is suggestive that poor women may require suitable approaches in reaching them with information on the array of services covered by NHIS. On account of the high fertility among the poor [37], intensifying efforts to communicate to poor women that malaria treatment is covered by NHIS can save more mothers and newborns [38].

Women who were subscribed to the NHIS had higher odds of reporting that NHIS covers malaria treatment. Past empirical evidence indicates that women who are not subscribed to NHIS mostly resort to self-medication or informal healthcare due to high cost of healthcare [39]. Meanwhile, NHIS subscribers may be more health conscious and therefore inquire about all the benefits that are accessible to them under the NHIS. The NHIS has operated over one and half decade since 2005 [12]. However, subscription rate is below 40% [14]. Sensitising women on the range of services covered by the NHIS may be an essential strategy to enhance subscription and improve maternal health.

Women in the Savanna zone had higher odds of indicating that NHIS covers malaria treatment. The Savanna Zone of Ghana covers the northern parts of the country [40]. Compared to the south, northern Ghana is characterized by limited healthcare facilities and health personnel [41, 42]. The Savanna zone is the poorest in the country [43, 44] and experiences highest seasonal malaria transmission and malaria among under five children [27, 31, 45]. The Savanna zone also bears the highest fertility rate in the country [46]. Based on these factors, it is expected that women in the Savanna zone will have more contacts with the health system and therefore have higher chance of being informed by health workers that malaria is covered by health insurance.

## Strengths and limitations

The study has a number of strengths. The study used nationally representative data, making the findings generalisable to all women aged 15-49 in the country. The rigour of the analytical procedure also strengthens the reliability and validity of the findings. The study, however, has some notable shortfalls. This is a cross-sectional study, thus causal relationships between the independent variables and the outcome variable cannot be established. Since the data used were self-reported, there is the possibility of both recall and social desirability biases.

## Conclusion

This study has shown that individual, community and regional level factors affect women’s knowledge on whether NHIS covers malaria care or not. Individual and community level interventions to enhance women’s knowledge on this should utilise mass media outlet like the radio. This will help to publicise to the poor and the uneducated women that NHIS covers malaria as these women had lower odds of knowing that NHIS covers malaria treatment. Educational interventions should be targeted at women with no or limited education, poorest women and women who are not subscribed to NHIS. This could persuade those who are not yet subscribed to enroll. The educational interventions can also sustain the interest of women who are already subscribed in order for them not to default NHIS renewal.

## Data Availability

Data used for the study is freely available to the public; https://dhsprogram.com/data/available-datasets.cfm.

## Abbreviatons

ANC: Antenatal Care
aOR: adjusted Odds Ratio
CI: Confidence interval
CrI: Credible interval
DIC: Deviance Information Criterion
EA: Enumeration areas
GDHS: Ghana Demographic and Health Survey
GSS: Ghana Statistical Service
ICF: Inner-City Fund
IpTP: Intermittent Preventive Treatment in Pregnancy
IRB: Institutional Review Board
MCMC: Markov Chain Monte Carlo
MOR: Median Odds Ratio
NHIA: National Health Insurance Authority
NHIS: National Health Insurance Scheme
OR: Odds Ratio
PHC: Population and Housing Census
PNC: Postnatal Care
SSA: sub-Saharan Africa
VIF: Variance Inflation Factor
VPC: Variance Partition Coefficient
